# Association of Childhood History of Parental Incarceration and Juvenile Justice Involvement With Mental Health in Early Adulthood

**DOI:** 10.1001/jamanetworkopen.2019.10465

**Published:** 2019-09-04

**Authors:** Nia Heard-Garris, Kaitlyn Ann Sacotte, Tyler N. A. Winkelman, Alyssa Cohen, Patricia O. Ekwueme, Elizabeth Barnert, Mercedes Carnethon, Matthew M. Davis

**Affiliations:** 1 Department of Pediatrics, Northwestern University Feinberg School of Medicine, Chicago, Illinois; 2 Division of Academic General Pediatrics, Department of Pediatrics, Ann & Robert H. Lurie Children’s Hospital of Chicago, Chicago, Illinois; 3 Mary Ann & J. Milburn Smith Child Health Research, Outreach, and Advocacy Center, Stanley Manne Children’s Research Institute, Ann & Robert H. Lurie Children’s Hospital of Chicago, Chicago, Illinois; 4 Division of General Internal Medicine, Department of Medicine, Hennepin Healthcare, Minneapolis, Minnesota; 5 Hennepin Healthcare Research Institute, Minneapolis, Minnesota; 6 Department of Pediatrics, Ann & Robert H. Lurie Children’s Hospital of Chicago, Chicago, Illinois; 7 Department of Pediatrics, David Geffen School of Medicine at UCLA, Los Angeles, California; 8 Department of Preventive Medicine, Northwestern University Feinberg School of Medicine, Chicago, Illinois; 9 Department of Medicine, Northwestern University Feinberg School of Medicine, Chicago, Illinois; 10 Department of Medical Social Sciences, Northwestern University Feinberg School of Medicine, Chicago, Illinois

## Abstract

**Importance:**

Young adults with a childhood history of parental incarceration (PI) or juvenile justice involvement (JJI) are more likely to have worse mental health outcomes than their peers. However, the association between mental health and exposure to both PI and JJI (PI plus JJI) is unclear.

**Objective:**

To determine the association of PI plus JJI exposure with mental health outcomes in young adulthood.

**Design, Setting, and Participants:**

A cross-sectional study of the US National Longitudinal Survey of Adolescent to Adult Health was conducted to examine the associations between PI, JJI, and PI plus JJI and mental health outcomes (ie, depression, anxiety, posttraumatic stress disorder, suicidal ideation, and mental health counseling). In-home interviews were conducted of 13 083 participants; 704 participants with PI after age 18 years were excluded, and 12 379 participants formed the analysis sample. Participants were in grades 7 to 12 in 1994 to 1995 and were ages 24 to 32 years at follow-up in 2008. Data analysis was completed in 2019.

**Exposures:**

Parental incarceration, JJI, or PI plus JJI before age 18 years.

**Main Outcomes and Measures:**

Mental health outcomes in early adulthood (ages 24-32 years). The analysis included multivariable logistic regression models; accounted for individual, family, and geographic-level factors; and generated adjusted odds ratios.

**Results:**

Among 13 083 participants (6962 female; weighted proportion, 49.6%) with a mean age at wave 1 of 15.4 years (95% CI, 15.2-15.7 years), 10 499 (80.2%) did not have a history of PI or JJI, 1247 (9.1%) had childhood PI, 704 (5.2%) had PI after age 18 years, 492 (4.5%) had JJI only, and 141 (1.2%) had PI plus JJI. Sociodemographic characteristics varied by exposure. Exposure to both PI and JJI was associated with a greater risk of depression (adjusted odds ratio, 2.80; 95% CI, 1.60-4.90), anxiety (adjusted odds ratio, 1.89; 95% CI, 1.08-3.31), and posttraumatic stress disorder (adjusted odds ratio, 2.92; 95% CI, 1.09-7.82) compared with peers with neither exposure. Exposure to both PI and JJI did not have an additive association with mental health beyond PI or JJI alone.

**Conclusions and Relevance:**

This study suggests that exposure to the criminal justice system during childhood places individuals at risk for poor mental health outcomes in early adulthood. Clinical, advocacy, and policy efforts that prioritize reducing the impact of the US criminal justice system on children may yield substantive improvements in the mental well-being of those individuals as adults.

## Key Points

**Question:**

Is a childhood history of parental incarceration and juvenile justice involvement associated with mental health conditions in early adulthood?

**Findings:**

In this nationally representative cross-sectional study, young adults with a history of both parental incarceration and juvenile justice involvement reported more mental health conditions compared with peers with no justice system exposure during childhood.

**Meaning:**

Parental incarceration and juvenile justice involvement may be associated with mental illness in young adult populations.

## Teaser

This cross-sectional study examines whether a childhood history of exposure to parental incarceration and/or juvenile justice involvement is associated with mental health outcomes in early adulthood.

## Introduction

Approximately 6.6 million individuals are under the supervision of the US adult correctional system.^1^ As a result, 5 million US children have had a resident parent incarcerated.^2^ Parental incarceration (PI) has long-term, negative health consequences that are independent of underlying racial/ethnic and socioeconomic disparities.^2,3,4,5,6^ Children of incarcerated adults have been shown to have significantly worse health behaviors, health outcomes, and health care utilization patterns in adulthood compared with their peers.^7,8,9,10,11,12^ For example, individuals affected by PI are more likely to smoke cigarettes, abuse alcohol or prescription drugs, and engage in high-risk sexual behaviors, while also forgoing medical care.^8^ Similarly, PI and mental health are closely associated. Young adults with PI histories have higher rates of depression, anxiety, and posttraumatic stress disorder (PTSD) than those without histories of PI.^7^

Parental incarceration is also strongly associated with juvenile justice involvement (JJI). Youth affected by PI are estimated to be involved in the juvenile justice system at 3 times the rate of their peers without PI.^13^ In 2017, more than 800 000 US youth younger than 18 years were arrested,^14^ and 53 000 were held in correctional facilities.^15^ Juvenile justice involvement is independently associated with poor health outcomes into adulthood.^16,17,18,19,20^ Similar to the association of PI, youth with JJI have increased rates of depression, anxiety, panic disorder, and substance abuse.^16,21^ Youth affected by PI or JJI also may have lower income, lower rates of employment, and higher rates of substance abuse than their counterparts in young adulthood.^22,23^ Thus, justice system exposure creates psychosocial and health risks that become intergenerational and span the life course.^13^

Although the association between PI and subsequent JJI has been established, national data on the frequency with which young adults are exposed to both types of criminal justice involvement are limited. Furthermore, little is known about the long-term mental health of this dually vulnerable population. Adverse childhood experiences, such as PI, have a cumulative association with health outcomes in adulthood.^3,4,6,24,25,26,27^ However, much of the current health research focuses on PI or JJI individually, largely ignoring the intergenerational cycle of incarceration in the United States. The cumulative influence of PI and JJI (PI plus JJI) on subsequent adult mental health reflects a critical knowledge gap.

This study aims to describe the population affected by PI plus JJI and to identify the association between mental illness and PI plus JJI. We hypothesized that adolescents who experienced the combination of PI plus JJI would have higher rates of mental illness in young adulthood than youth who experienced neither, or either in isolation. By focusing on this unique group, we may better inform public health and clinical practice, as well as criminal justice and juvenile justice policy and practice.

## Methods

### Study Design

We used data from the National Longitudinal Study of Adolescent Health to Adult Health (Add Health, 1994-2008)^28^ to conduct a retrospective, cross-sectional study to examine the association between PI, JJI, and PI plus JJI and mental health outcomes. This study was approved by the Ann & Robert H. Lurie Children’s Hospital institutional review board. Add Health participants provided written informed consent in accordance with the University of North Carolina School of Public Health institutional review board guidelines that are based on 45CFR46. Additional information on the sampling procedures and study design is documented elsewhere.^28^ This study follows the Strengthening the Reporting of Observational Studies in Epidemiology (STROBE) reporting guideline.

### Data and Study Population

Add Health^28^ was a nationally representative longitudinal study that followed adolescents from high schools in the United States into adulthood. Junior high and high schools (grades 7-12) served as the primary sampling unit for the clustered study design. In this study, we used data from both waves I and IV of Add Health. Wave I was initiated during the 1994 to 1995 school year, during which 18 924 adolescents (aged 12-19 years) completed in-home interviews and were invited to participate in follow-up waves. Wave IV included in-home interviews that were completed in 2008. Wave IV included 13 861 respondents (aged 24-32 years) from wave I with a valid sample weight.

The analytic sample included 12 379 participants with complete data for both wave I and wave IV (13 083 [94.4%] of wave IV participants), excluding 704 (5.2%) individuals reporting a history of PI after age 18 years to examine the associations of criminal justice exposure in childhood separate from PI exposure after childhood.

### Measures

#### Exposure to Parental Incarceration

Respondents were asked in wave IV whether their biological mother or father ever spent time in jail or prison and their ages at both the first and most recent time their biological mother or father was in jail or prison. Individuals who reported any history of PI from birth to before age 18 years were included in the PI exposure group. Individuals who reported a history of PI after age 18 years were excluded to examine the associations of criminal justice exposure in childhood.

#### Exposure to Juvenile Justice Involvement

In wave IV, participants were asked whether they had ever been convicted of or pled guilty to a crime or been found delinquent in juvenile court and their age the first time they were incarcerated in a jail, prison, juvenile detention center, or correctional facility. They were asked to report the number of times they were arrested before their 18th birthday. Individuals who reported any history of arrest, conviction, pleading guilty, or being found delinquent in juvenile court before age 18 years were considered to have a history of JJI. In addition, any individual who reported being incarcerated in jail, prison, juvenile detention center, or correctional facility before age 18 years was included in the JJI exposure group.

#### Mental Health Outcomes

Data on adult mental health were collected in wave IV. Respondents were asked whether they had ever received a diagnosis of anxiety, depression, or PTSD. In addition, they were asked whether they had received psychological or emotional counseling within the last year, experienced suicidal ideation within the last year, or had attempted suicide within the last year. Respondents were designated to have 1 of the aforementioned mental health conditions if they responded “yes” to any of these questions.

#### Covariates

We controlled for sociodemographic factors from wave I. The factors included sex, race/ethnicity, age, family structure (2-parent, 1-parent, or no-parent households), parental education (mother and father), receipt of public assistance, and geographic classification of residence (urban, suburban, or rural).^7,8,29^

### Statistical Analysis

 Data analysis was completed in 2019. We summarized sociodemographic characteristics of the study population by 3 exposure groups: PI only, JJI only, and PI plus JJI. The analytic sample was restricted to participants who had complete sociodemographic and exposure data (94.4% of wave IV respondents). We used logistic regression models to examine associations between the 3 exposure groups and mental health outcomes. In adjusted multivariable models, we controlled for the aforementioned covariates. We calculated 3 measures to evaluate interaction—relative excess risk due to interaction, attributable proportion due to interaction, and synergy index—and their 95% confidence intervals to test for additive interaction of combined PI and JJI exposures. Relative excess risk due to interaction estimates additive interactions in case-control study designs. Attributable proportion due to interaction refers to the proportion of disease that is due to interaction among persons with both exposures. Synergy index measures whether the risk ratio for both exposures together is greater than the sum of each of the risk ratios considered separately.^30^ All analyses were conducted using Stata statistical software version 14.0 (StataCorp), accounting for the clustered, stratified survey design, and used survey weights (provided with the data set) to generate national population estimates unless otherwise noted.

## Results

The 13 083 participants (6962 female; weighted proportion, 49.6%) had a mean age at wave 1 of 15.4 years (95% CI, 15.2-15.7 years). Table 1 presents the weighted sociodemographic characteristics of our sample by history of PI and JJI: 10 499 (80.2%) did not have a history of PI or JJI, 1247 (9.1%) had childhood PI exposure only, 704 (5.2%) had PI after age 18 years, 492 (4.5%) had JJI exposure only, and 141 (1.2%) had PI plus JJI exposure. The analytic sample included 12 379 individuals with no history of PI or JJI or a history of justice exposure before age 18 years. Non-Hispanic black individuals had a disproportionately high prevalence of criminal justice exposure, accounting for 23.8% (95% CI, 17.8%-31.1%) of those who reported childhood PI only, 20.0% (95% CI, 12.9%-29.7%) of those who reported JJI only, 33.1% (95% CI, 21.9%-46.7%) of those who reported both PI and JJI, contrasting with 12.9% (95% CI, 9.6%-16.9%) who reported neither PI nor JJI. Hispanic participants were also disproportionately represented among those with PI plus JJI, accounting for 17.6% (95% CI, 10.1%-28.8%) of that group but only 11.3% (95% CI, 8.2%-15.4%) without PI or JJI. Notably, individuals with PI plus JJI histories lived in households with either 1 parent (67.4%; 95% CI, 56.5%-76.7%) or no biological parents present (12.2%; 95% CI, 7.1%-20.3%) and were more likely than other individuals to receive public assistance (30.0%; 95% CI, 20.2%-42.1%). Of individuals with both PI plus JJI exposures, 54.2% (95% CI, 43.1%-64.8%) were from urban environments, compared with 27.1% (95% CI, 22.8%-31.9%) among individuals with neither PI nor JJI exposure.

**Table 1.** Characteristics of the 12 379 Participants in the Analytic Sample, by Criminal Justice Exposure Before Age 18 Years, National Longitudinal Study of Adolescent to Adult Health

Characteristic	Weighted % (95% CI)^a^			
	No PI or JJI (n = 10 499)	PI Only (n = 1247)	JJI Only (n = 492)	Both PI and JJI (n = 141)
Female	51.4 (50.0-52.9)	56.5 (52.4-60.6)	12.3 (9.2-16.2)	18.4 (12.1-26.9)
Race/ethnicity				
Non-Hispanic white	68.8 (62.9-74.2)	57.8 (50.4-64.8)	59.0 (49.1-68.3)	43.0 (31.3-55.6)
Non-Hispanic black	12.9 (9.6-16.9)	23.8 (17.8-31.1)	20.0 (12.9-29.7)	33.1 (21.9-46.7)
Hispanic	11.3 (8.2-15.4)	13.1 (9.6-17.6)	12.2 (8.1-18.0)	17.6 (10.1-28.8)
Non-Hispanic, other	7.0 (5.4-9.1)	5.4 (3.7-7.7)	8.7 (5.7-13.2)	6.3 (2.4-15.7)
Age, mean (95% CI), y^b^	15.5 (15.3-15.7)	15.2 (14.9-15.5)	15.2 (14.8-15.5)	15.2 (14.7-15.7)
Family structure				
2-Parent household	66.3 (64.2-68.4)	26.2 (22.7-29.9)	42.6 (37.4-48.0)	20.3 (12.0-32.3)
1-Parent household	30.9 (30.7-34.5)	65.1 (61.6-68.4)	53.7 (48.0-59.3)	67.4 (56.5-76.7)
No biological parents in household	2.8 (2.2-3.4)	8.7 (6.7-11.3)	3.7 (1.9-7.0)	12.2 (7.1-20.3)
Mother’s education				
No resident mother	3.3 (2.8-3.8)	5.5 (4.1-7.5)	7.4 (4.6-11.6)	11.0 (5.9-19.8)
Less than high school	16.2 (14.0-18.8)	24.6 (20.9-28.6)	17.8 (12.9-23.9)	31.3 (21.9-42.5)
High school, trade school, or general equivalency diploma	41.3 (38.6-44.1)	45.0 (40.7-49.5)	41.8 (35.7-48.1)	41.5 (31.4-52.4)
College education or higher	39.2 (35.9-42.6)	24.9 (20.9-29.3)	33.0 (26.2-40.8)	16.2 (9.1-27.0)
Father’s education				
No resident father	20.8 (19.0-22.9)	47.3 (43.6-50.9)	37.2 (31.2-43.7)	51.3 (39.2-63.3)
Less than high school	13.5 (11.5-15.7)	18.2 (15.1-21.7)	12.2 (9.0-16.4)	21.0 (13.2-31.9)
High school, trade school, or general equivalency diploma	30.4 (27.9-33.1)	24.8 (21.3-28.6)	28.5 (23.3-34.2)	20.8 (12.3-32.9)
College education or higher	35.2 (31.8-38.8)	9.8 (7.8-12.3)	22.1 (16.7-28.6)	6.9 (2.9-15.3)
Received public assistance	7.3 (6.0-8.7)	24.1 (20.9-27.7)	12.0 (7.3-19.2)	30.0 (20.2-42.1)
Geographic classification of residence				
Suburban	40.7 (35.4-46.2)	30.1 (24.9-35.8)	37.1 (29.9-45.0)	24.4 (16.1-35.1)
Urban	27.1 (22.8-31.9)	37.9 (31.2-45.2)	37.7 (29.1-47.2)	54.2 (43.1-64.8)
Rural	29.1 (24.3-34.3)	28.2 (21.8-35.5)	23.1 (17.0-30.7)	18.5 (11.2-28.8)
Other	3.2 (2.3-4.3)	3.8 (2.6-5.6)	2.0 (0.9-4.8)	3.0 (0.9-10.0)

Abbreviations: JJI, juvenile justice involvement; PI, parental incarceration.

^a^ Reflects the representative proportion in the target US population. Percentages may not total 100% because of rounding.

^b^ Age at wave I (1994-1995).

Table 2 displays the prevalence of mental health outcomes among our analytic sample by criminal justice exposure. In general, individuals without PI or JJI exposure had a lower prevalence of all the mental health outcomes examined compared with their peers exposed to PI and/or JJI. Notably, young adults with PI only had a higher prevalence of depression (23.7%; 95% CI, 20.0%-27.9%), anxiety (18.5%; 95% CI, 15.4%-22.0%), and suicidal thoughts (10.0%; 95% CI, 8.0%-12.5%), whereas those with JJI only had the highest prevalence of PTSD (6.1%; 95% CI, 3.8%-9.6%) and mental health counseling (14.3%; 95% CI, 10.6%-18.9%). Individuals with PI plus JJI had a somewhat higher prevalence of suicide attempts (3.0%; 95% CI, 0.9%-9.6%). In addition, 22.2% (95% CI, 14.7%-32.1%) of young adults with PI plus JJI reported depression and 12.4% (95% CI, 6.6%-22.3%) reported receiving mental health counseling.

**Table 2.** Prevalence of Mental Health Outcomes for 12 379 Participants in the Analytic Sample, by Criminal Justice Exposure Before Age 18 Years, National Longitudinal Study of Adolescent to Adult Health at Wave I

Mental Health Outcomes	Weighted % (95% CI)^a^			
	No PI or JJI (n = 10 499)	PI Only (n = 1247)	JJI Only (n = 492)	Both PI and JJI (n = 141)
Depression	14.5 (13.2-15.8)	23.7 (20.0-27.9)	20.8 (16.4-26.1)	22.2 (14.7-32.1)
Anxiety	12.3 (11.2-13.4)	18.5 (15.4-22.0)	13.0 (9.9-17.0)	13.5 (8.0-21.8)
Posttraumatic stress disorder	2.3 (1.9-2.8)	4.2 (2.9-5.9)	6.1 (3.8-9.6)	5.6 (2.3-12.9)
Suicidal thoughts^b^	6.2 (5.5-6.9)	10.0 (8.0-12.5)	7.8 (5.1-11.7)	9.0 (4.2-18.3)
Suicide attempt^b^	1.3 (0.9-1.9)	1.8 (1.1-3.0)	2.8 (1.3-6.0)	3.0 (0.9-9.6)
Mental health counseling^b^	8.9 (8.1-9.7)	12.7 (10.3-15.5)	14.3 (10.6-18.9)	12.4 (6.6-22.3)

Abbreviations: JJI, juvenile justice involvement; PI, parental incarceration.

^a^ Reflects the representative proportion in the target US population.

^b^ Within the last 12 months.

### Unadjusted Analyses

In unadjusted analyses, individuals with histories of PI, JJI, or PI plus JJI had higher odds of adverse mental health outcomes compared with individuals with no PI or JJI history (Table 3). Specifically, all 3 childhood criminal justice exposures were associated with depression (unadjusted odds ratios [ORs], 1.84 [95% CI, 1.45-2.34] for PI only, 1.56 [95% CI, 1.14-2.12] for JJI only, and 1.69 [95% CI, 1.01-2.84] for PI and JJI).

**Table 3.** Unadjusted ORs of Adult Mental Health Outcomes for and Mental Health Care Utilization by 1880 Participants Who Experienced Criminal Justice Exposure Before Age 18 Years

Mental Health Outcomes	Unadjusted OR (95% CI)		
	PI Only	JJI Only	Both PI and JJI
Depression	1.84 (1.45-2.34)^a^	1.56 (1.14-2.12)^b^	1.69 (1.01-2.84)^c^
Anxiety	1.62 (1.29-2.04)^a^	1.07 (0.78-1.49)	1.12 (0.63-1.97)
Posttraumatic stress disorder	1.84 (1.21-2.78)^b^	2.72 (1.66-4.48)^a^	2.48 (0.98-6.33)
Suicidal thoughts^d^	1.70 (1.32-2.18)^a^	1.28 (0.80-2.04)	1.52 (0.67-3.45)
Suicide attempt^d^	1.36 (0.73-2.51)	2.12 (0.89-5.04)	2.29 (0.63-8.29)
Mental health counseling^d^	1.50 (1.16-1.93)^b^	1.72 (1.22-2.41)^b^	1.46 (0.72-2.98)

Abbreviations: JJI, juvenile justice involvement; OR, odds ratio; PI, parental incarceration.

^a^ *P* < .001.

^b^ *P* < .01.

^c^ *P* < .05.

^d^ Within the last 12 months.

Histories of PI only and JJI only were associated with PTSD (unadjusted ORs, 1.84 [95% CI, 1.21-2.78] for PPI only and 2.72 [95% CI, 1.66-4.48] for JJI only) and the receipt of mental health counseling (unadjusted ORs, 1.50 [95% CI, 1.16-1.93] for PPI only and 1.72 [95% CI, 1.22-2.41] for JJI only). Parental incarceration only was associated with anxiety (unadjusted OR, 1.62; 95% CI, 1.29-2.04) and suicidal thoughts (unadjusted OR, 1.70; 95% CI, 1.32-2.18), whereas JJI and PI plus JJI were not.

### Adjusted Analyses

Individuals with any history of childhood justice system exposure had significantly worse mental health outcomes than their peers without histories of PI or JJI (Table 4). Individuals exposed only to PI in childhood had higher odds of depression (adjusted OR, 1.88; 95% CI, 1.44-2.45) and anxiety (adjusted OR, 1.73; 95% CI, 1.35-2.20) compared with individuals without childhood PI or JJI exposure. In addition, individuals with PI exposure were more likely to have received mental health counseling (adjusted OR, 1.65; 95% CI, 1.26-2.17). Suicidal thoughts (adjusted OR, 1.55; 95% CI, 1.19-2.02), but not suicide attempts, were also significantly more likely in this population. Exposure to JJI alone was also associated with higher odds of depression (adjusted OR, 2.53; 95% CI, 1.79-3.59), anxiety (adjusted OR, 1.72; 95% CI, 1.20-2.45), PTSD (adjusted OR, 3.50; 95% CI, 2.11-5.81), and suicide attempts (adjusted OR, 2.89; 95% CI, 1.27-6.60) compared with individuals without childhood PI or JJI exposure and also was associated with higher odds of receiving mental health counseling (adjusted OR, 2.34; 95% CI, 1.64-3.32).

**Table 4.** Adjusted ORs of Adult Mental Health Outcomes for and Mental Health Care Utilization by 1880 Participants Who Experienced Criminal Justice Exposure Before Age 18 Years

Mental Health Outcomes	Adjusted OR (95% CI)^a^		
	PI Only	JJI Only	Both PI and JJI
Depression	1.88 (1.44-2.45)^b^	2.53 (1.79-3.59)^b^	2.80 (1.60-4.90)^b^
Anxiety	1.73 (1.35-2.20)^b^	1.72 (1.20-2.45)^c^	1.89 (1.08-3.31)^d^
Posttraumatic stress disorder	1.52 (0.95-2.43)	3.50 (2.11-5.81)^b^	2.92 (1.09-7.82)^d^
Suicidal thoughts^e^	1.55 (1.19-2.02)^b^	1.28 0.81-2.02)	1.44 (0.63-3.30)
Suicide attempt^e^	1.19 (0.71-2.00)	2.89 (1.27-6.60)^c^	2.50 (0.67-9.36)
Mental health counseling^e^	1.65 (1.26-2.17)^b^	2.34 (1.64-3.32)^b^	2.08 (1.01-4.27)^d^

Abbreviations: JJI, juvenile justice involvement; OR, odds ratio; PI, parental incarceration.

^a^ Data were adjusted for sociodemographic factors from wave I, including sex, race/ethnicity, age, family structure (2-parent, 1-parent, or no-parent households), parental education (mother and father), receipt of public assistance, and geographic classification of residence (urban, suburban, or rural).^7,8,29^

^b^ *P* < .001.

^c^ *P* < .01.

^d^ *P* < .05.

^e^ Within the last 12 months.

Individuals with PI plus JJI exposure had the highest odds of depression (adjusted OR, 2.80; 95% CI, 1.60-4.90), anxiety (adjusted OR, 1.89; 95% CI, 1.08-3.31), and PTSD (adjusted OR, 2.92; 95% CI, 1.09-7.82) compared with individuals with no childhood PI or JJI. These individuals also were more likely to have received mental health counseling compared with those without PI or JJI histories (adjusted OR, 2.08; 95% CI, 1.01-4.27).

For each mental health outcome, we used relative excess risk due to interaction, attributable proportion due to interaction, and synergy index to test for additive interaction. None of the outcomes demonstrated a statistically significant additive interaction (results not shown).

## Discussion

In this analysis of a nationally representative sample, a consistent association exists between combined PI and JJI exposures in childhood and subsequent adverse mental health outcomes in early adulthood. Our findings suggest that dual exposure (PI plus JJI) in childhood is associated with increased risk of depression, anxiety, and PTSD in young adulthood. Although childhood exposure to both PI and JJI may not contribute to additional risk, there may be differential susceptibility to adverse mental health outcomes depending on the type of justice exposure experienced during childhood.

Previous studies^7,16,21,31^ have shown that PI alone and JJI alone are independently associated with worse mental health outcomes. Adults who were exposed to PI during childhood are more likely than their peers without exposure to PI to have anxiety, depression, and PTSD.^7^ Our results support those previous findings and add that the young adults who experience childhood PI are more likely to experience thoughts of suicide compared with those with no PI or JJI history. In other samples,^21,31,32^ individuals with JJI exposure were also more likely to have substance abuse disorders, mood disorders, anxiety, depression, and sexually transmitted infections compared with peers. Our mental health findings are consistent with the extant studies and also indicate that individuals with JJI are more likely to experience PTSD and report suicide attempts compared with peers without PI or JJI.

Our analyses highlight that a history of combined PI and JJI during childhood, although less common than either PI or JJI alone, occurs for 1 in 100 US children overall (1.2%) and is disproportionately more common among youth of color (Table 1). Parental incarceration plus JJI was associated with the highest odds of depression and anxiety in adulthood in this sample. Posttraumatic stress disorder was also associated with the dual exposure group, with the odds slightly lower than those for JJI only. The associations of PI plus JJI with depression, anxiety, and PTSD did not appear to be additive above PI or JJI exposure alone. However, certain mental health outcomes did appear to be associated with a particular type of justice exposure. For example, in the adjusted models, suicidal thoughts were associated with PI only, and suicide attempts were associated with JJI only.

Interestingly, the JJI only and PI plus JJI groups had the highest odds of receiving mental health counseling within the last year. Receipt of counseling could be a reassuring sign that at-risk adults are receiving needed mental health treatment or could also signify that adults most in need of counseling had accessed such services. Many individuals in this population have public insurance coverage, which may diminish cost as a barrier to receiving mental health care.^29,33^ In addition, contact with the justice system may connect individuals more directly with mental health resources compared with those who might not interface with the system. Still, with 22.2% of young adults with PI plus JJI reporting depression and 12.4% receiving mental health counseling, there still may be unmet mental health needs in this population. This may explain the higher odds of suicide attempts in the population exposed to JJI compared with the individuals without PI or JJI exposure. This finding, along with the paucity of data regarding the quality, frequency, and reason for the counseling make it difficult to determine whether this population of young adults at-risk for mental illness is receiving adequate mental health care.

### Limitations

This study must be interpreted within the context of its limitations. First, the unweighted sample of individuals with PI plus JJI was small and focused exclusively on biological parents. However, it is one of a few nationally representative data sets that incorporate PI, JJI, and long-term health outcomes. Our focus on the incarceration of biological parents in childhood may not fully account for young adults who experienced the incarceration of nonbiological caregivers, those who had limited relationships with their biological parents, or those who experienced the incarceration of their biological parents after age 18 years. In addition, both mental health diagnoses and incarceration are sensitive topics that may be underreported because of social desirability bias or shame,^34^ which may lead to underestimates of effect sizes in the associations we report or affect the nature of the findings. Also, although single-item self-reports for suicidal ideation and attempts within the past year reduce recall biases, they limit our understanding of the lifetime risk. Furthermore, our findings relied on self-reported mental health diagnoses, which could have been underreported. Also, the diagnoses that were made by different health care professionals may have applied the diagnostic criteria differently. However, it is unclear how this would influence the estimates within the PI, JJI, and PI plus JJI exposure groups, given that they were more likely to have received mental health counseling than their peers with neither PI nor JJI. In addition, because of the cross-sectional study design drawing on data collected at wave IV, neither causal inference nor the directionality of the association can be confirmed. Mental health conditions, in addition to adverse childhood experiences, may predispose individuals to justice system involvement, which, in turn, may make them more likely to be incarcerated in a juvenile detention center.^35^ This study did not account for either the type of offense contributing to JJI or PI^8^ or the duration or frequency of the justice system exposure. These facets of PI and JJI were beyond the scope of this study but should be considered for future studies, because they may further explain the observed associations.

### Implications

Individuals who experience PI and JJI during childhood have an increased risk of mental health concerns in adulthood. These associations suggest that policy makers should consider the multiple avenues through which children are affected by the criminal justice system and the intergenerational health risks such contact perpetuates. These findings also generate additional questions as to the timing or emergence of mental health conditions in this group. Previous research has suggested that children with PI have evidence of behavioral health concerns but not diagnosed mental health problems during childhood.^34,36^ In addition, PI may represent an important opportunity for policy-level intervention, because previous studies have shown that PI may have a stronger influence on children than other types of parental absence.^37^ Future research that examines the influence of PI on bonding and attachment and those aspects of childhood development associated with JJI involvement, as well as future mental health outcomes, would further explicate the intergenerational connection between PI and JJI. Furthermore, although PI and JJI are linked, currently, PI exposure is considered an adverse childhood experience and JJI is not.^3^ Given the association of JJI with adverse mental health outcomes in adulthood in this study, perhaps JJI should be considered an adverse childhood experience going forward.

Future studies should evaluate whether pediatric screening for criminal justice–related exposures followed by early mental health interventions may mitigate some of the precursors for adult mental health conditions. In particular, integrating routine screening for PI and JJI within the typical health care visit for youth may reduce stigma. Screening can also allow health care professionals who focus on children and young adults to refer families for additional services for unmet needs, such as housing and food insecurity, which are known to disproportionately affect this justice-involved population and also may have implications for adult mental health.^36^ In addition, examining the benefits of the integration of legal services within the pediatric medical home, such as medical-legal partnerships, may aid clinicians and families in navigating associated unmet social, health, and educational needs. These findings also suggest that such approaches may be beneficial into adulthood.

It is important to note that pediatricians and other health care professionals can screen and treat only those who receive health care, which suggests that additional public health approaches beyond clinic-based services are needed. Juvenile detention centers could help link adolescents to either new or their established primary care homes after incarceration. For youth experiencing PI, the courts might consider the impact of PI on children and take parental status into account during sentencing. Parents and their children jointly experiencing justice involvement may benefit from support services to help address additional needs that arise during incarceration and likely reverberate beyond the end of a sentence.

## Conclusions

The findings from this nationally representative sample of community-dwelling adults suggest that the negative influences of criminal justice exposure during childhood are evident into adulthood. Clinicians, public health professionals, the justice system, and policy makers are critical stakeholders in addressing the effects of incarceration on children, adolescents, and adults. Developing and implementing alternatives to incarceration in the juvenile and adult criminal justice systems may be an important step to ameliorating adverse mental health outcomes for youth exposed to the justice system that are evident in their lives as adults. Future studies should continue to focus on populations affected by PI and JJI, including understanding the association of this dual exposure with physical health and subsequent corresponding disparities.
